# 改性松香键合二氧化硅高效液相色谱固定相的制备及其对三七总皂苷的分离

**DOI:** 10.3724/SP.J.1123.2021.07008

**Published:** 2022-03-08

**Authors:** Wenbo XIE, Lu XIA, Hao LI, Wen LI, Yu CAO, Yun HUANG, Fuhou LEI

**Affiliations:** 1.广西民族大学化学化工学院, 林产化学与工程国家民委重点实验室, 广西林产化学与工程重点实验室/协同创新中心, 广西 南宁 530006; 1. Key Laboratory of Chemistry and Engineering of Forest Products, State Ethnic Affairs Commission, Guangxi Key Laboratory of Chemistry and Engineering of Forest Products, Guangxi Collaborative Innovation Center for Chemistry and Engineering of Forest Products, College of Chemistry and Chemical Engineering, Guangxi University for Nationalities, Nanning 530006, China; 2.湖北民族大学化学与环境工程学院, 湖北 恩施 445000; 2. School of Chemical and Environmental Engineering, Hubei Minzu University, Enshi 445000, China

**Keywords:** 松香键合固定相, 高效液相色谱, 三七总皂苷, rosin bonded stationary phase, high performance liquid chromatography (HPLC), *Panax notoginseng* saponins

## Abstract

三七中发挥药效的主要成分为三七皂苷R1、人参皂苷Rg1、人参皂苷Re、人参皂苷Rb1和人参皂苷Rd,用于贫血、冠心病、高血压、脑卒中后遗症等疾病的治疗,但其化学成分多且难分离。将氢化松香丙烯酸羟乙酯(HRHA)通过巯基-烯点击化学反应键合到烷基化硅胶表面,制备出一种新型的改性松香键合二氧化硅高效液相色谱固定相(SiO_2_@HRHA),用于三七总皂苷的分离。对色谱固定相进行一系列表征,表明SiO_2_@HRHA固定相具有球形度好和表面多孔的特点,比表面积为308.55 m^2^/g,平均孔径达到6.78 nm。将制备的固定相湿法装柱,色谱柱性能评价结果表明,SiO_2_@HRHA柱具备反相色谱行为、较好的流通性及重复性。从色谱柱的综合评价Tanaka测试可知,SiO_2_@HRHA柱具有较好的立体选择性及氢键容量。将其用于三七总皂苷的分离,SiO_2_@HRHA柱在一定程度上优于C18柱,而SiO_2_@HRHA柱的分离效果又优于实验室同期合成的色谱柱,对5种皂苷(R1、Rg1、Re、Rb1和Rd)的分离度依次为3.33、3.54、20.17和9.72,表明SiO_2_@HRHA高效液相色谱柱对三七总皂苷具有优异的分离效果,为从实际样品中分离纯化三七总皂苷提供了新思路。

## Abstract (en)

The sanqi is the dried root of *Panax notoginseng* (Burk.) F. H. Chen. The main components responsible for the drug actions of sanqi are notoginsenoside R1, ginsenoside Rg1, ginsenoside Re, ginsenoside Rb1, and ginsenoside Rd, which account for about 80% of the saponin content in sanqi. It is widely used in the treatment of anemia, coronary heart disease, hypertension, stroke sequelae, and other diseases. However, sanqi has many chemical components with complex and similar structures, which are difficult to separate. In this study, alkylated silica gel bonded with hydrogenated rosin hydroxyethyl acrylate (HRHA) was prepared via mercapto-ene click chemistry. A new type of modified rosin-bonded silica stationary phase (SiO_2_@HRHA) for high performance liquid chromatography was prepared for the separation of five saponins (R1, Rg1, Re, Rb1, and Rd). It was characterized by thermogravimetric analysis, Fourier-transform infrared spectroscopy, specific surface area and microporous physical adsorption and elemental analysis. The results showed that SiO_2_@HRHA had a regular spherical shape with porous surfaces, along with a specific surface area of 308.55 m^2^/g and an average pore diameter of 6.78 nm. Performance evaluation of the column revealed that the SiO_2_@HRHA column showed typical reversed-phase chromatographic behavior with better flowability and reproducibility. Results of the Tanaka test showed that SiO_2_@HRHA column had good stereoselectivity and hydrogen bond capacity. Compared to other stationary phases, e. g. silica modified with acrylopimaric acid (16-hydroxyethyl-34-hydroxyethyl acrylate) ester (AAE) and dihydroterpineol (DTP), which were prepared in our laboratory at the same time, the SiO_2_@HRHA column demonstrated better resolution (*R*_s_) for the separation of the five saponins under optimal chromatographic conditions. The *R*_s_ values for R1, Rg1, Re, Rb1, and Rd were 3.33, 3.54, 20.17 and 9.72, respectively on the SiO_2_@HRHA column. *R*_s_ between Rg1 and Re was also better than that obtained on a C18 column. *Panax notoginseng* saponins were separated on the SiO_2_@HRHA column using acetonitrile and water as the mobile phases at the flow rate of 1.0 mL/min at 25 ℃. The optimal UV detection wavelength was 203 nm. It was found that the five saponins could be separated better using the SiO_2_@HRHA column than the SiO_2_@AAE and SiO_2_@DTP columns. Because the ternary phenanthrene skeleton of the rosin group in SiO_2_@HRHA had structural similarity and good stereoselectivity to the polycyclic compounds (*Panax notoginseng* saponins). In addition, according to the hydrophobicity evaluation, the SiO_2_@HRHA column showed the best hydrophobicity among the three columns, which may be conducive to the separation of the five saponins. Thus, this study can provide a new avenue for the separation and purification of *Panax notoginseng* saponins from actual samples.

三七为五加科植物三七*Panax notoginseng* (Burk.) F. H. Chen的干燥根,发挥药效的主要成分是三七皂苷R1、人参皂苷Rg1、人参皂苷Re、人参皂苷Rb1和人参皂苷Rd,约占三七总皂苷含量的80%^[1]^。三七总皂苷既可以单独作为药物治疗疾病^[2]^,又可以与其他药物共同发挥药效^[3]^,具有较好的临床应用效果与药学开发价值^[4]^。但三七的化学成分多,结构繁杂且相似,较难分离。目前从三七中分离纯化皂苷类化合物需要经过大孔吸附树脂进行预处理,该过程繁琐且需要消耗大量有机试剂。高效液相色谱(HPLC)因其具有分析速度快、应用范围广的优点,广泛应用于食品、药品、环境污染物和生物样品等的分离^[5,6]^。高效液相色谱技术的关键是固定相,开发新型色谱固定相分离和纯化天然产物中的活性成分一直都是科学工作者的研究热点。因此,制备新型液相色谱固定相,用于三七总皂苷的高选择性分离和纯化具有重要意义。

松香可生物降解,绿色环保。本课题组制备的松香基高分子微球已应用于喜树碱^[7,8]^、紫杉醇^[9]^、多环芳烃^[10]^等的分离纯化。松香基高分子微球具有孔径丰富、孔结构可控以及与药物甾环结构相似的优势,但其粒径均一性差、比表面积小、机械强度低,不能满足色谱固定相的需求;而二氧化硅粒径均一,球形度好,比表面积大,机械强度高但是没有选择性。因此,本研究在前期工作的基础上将二氧化硅微球进行烷基化,引入新的反应位点,通过巯基-烯点击化学反应将松香基交联剂氢化松香丙烯酸羟乙酯(HRHA)键合到烷基化硅胶表面,得到改性松香键合硅胶高效液相色谱固定相(SiO_2_@HRHA)。通过热重、傅里叶变换红外光谱、比表面积及微孔物理吸附和有机元素分析等手段对色谱固定相进行表征;将制备的固定相湿法装柱,对色谱柱的类型、流通性及重复性进行评价,用Tanaka测试对比分析其立体选择性及氢键容量。最后将该色谱柱直接用于三七总皂苷的分离,为从实际样品中分离纯化三七总皂苷提供一种新思路。

## 1 实验部分

### 1.1 仪器与试剂

Ultra CP-24装柱机(超高压恒压泵,美国Scientific Systems公司); MAGNA-IR 550傅里叶变换红外光谱分析仪(美国Nicolet公司); STA449F3热重分析仪(德国Netzsch公司); ASAP2020比表面积及微孔物理吸附仪(美国Micromeritics公司); Vario EL cube有机元素分析仪(德国Elemantar公司); LC-15C型高效液相色谱仪(日本Shimadzu公司)。

氢化松香丙烯酸羟乙酯、丙烯海松酸(16-羟乙基-34-丙烯酸羟乙基)酯(AAE)(纯度95%,广西鼎弘树脂有限公司);二氢松油醇(DTP)(分析纯,成都金山化学试剂有限公司);硅胶(粒径:5 μm,比表面积:370 m^2^/g,苏州纳微科技股份有限公司); 3-巯基丙基三甲氧基硅烷(MAPS)(纯度95%),三七皂苷R1、人参皂苷Rg1、人参皂苷Re、人参皂苷Rb1和人参皂苷Rd(纯度≥98%),偶氮二异丁腈(AIBN)(纯度99%),均购于上海阿拉丁生化科技股份有限公司;乙腈(色谱纯,美国Fisher Scientific公司);其他试剂均为分析纯。

### 1.2 色谱固定相的制备

将5 g活化硅胶置于250 mL三口烧瓶中,加入50 mL无水甲苯、2 mL无水吡啶、5 mL硅烷偶联剂MAPS,于80 ℃下磁力搅拌8 h^[11]^,反应结束后冷却至室温,抽滤,收集固体,依次使用甲醇、丙酮和正己烷冲洗(每次200 mL,各洗2次),于60 ℃真空干燥12 h,制备出烷基化硅胶(SiO_2_-MAPS)。

将5 g烷基化硅胶、0.005 g偶氮二异丁腈、100 mL乙酸乙酯置于250 mL三口烧瓶内,同时添加3 g HRHA,于70 ℃下磁力搅拌反应8 h^[12]^,反应结束后,抽滤,收集固体,用无水乙醇洗涤(每次200 mL,洗涤4次),于80 ℃下真空干燥10 h,得到固定相SiO_2_@HRHA。合成路线如图1所示。

**图1:**
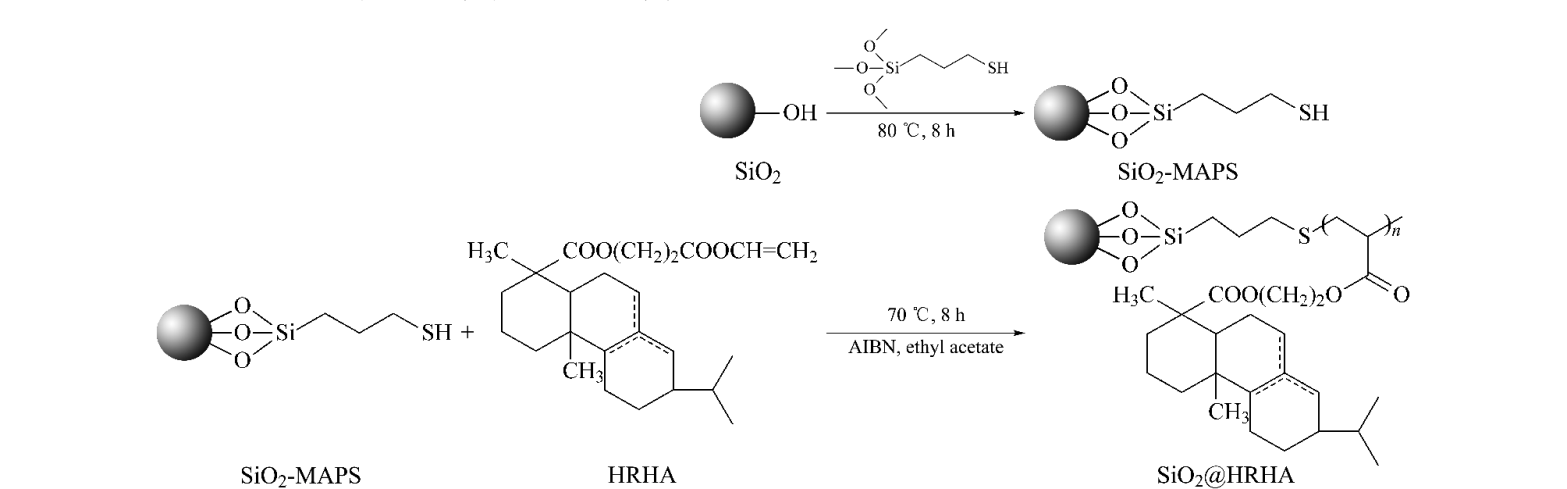
SiO_2_@HRHA的合成路线

将AAE和DTP在色谱固定相合成过程中分别替代氢化松香丙烯酸羟乙酯,制备出SiO_2_@AAE和SiO_2_@DTP固定相。

### 1.3 装柱

准确称量5.0 g固定相置于小烧杯内,加入58 mL甲醇(匀浆液)超声,使固定相分散均匀,放置备用。将不锈钢柱管(250 mm×4.6 mm)装到装柱机上,设置流速(24 mL/min)、装柱压力和时间(41 MPa, 20 min),将分散好的固定相倒入匀浆罐中,以甲醇为顶替液,进行装柱。

### 1.4 色谱条件

色谱柱为自制SiO_2_@HRHA柱(250 mm×4.6 mm);柱温25 ℃;流动相:A为乙腈,B为水,流速:1.0 mL/min。梯度洗脱程序:0~20 min, 18%A; 20~45 min, 18%A~46%A; 45~55 min, 46%A~55%A; 55~60 min, 55%A。进样量为20 μL;紫外检测波长203 nm。三七总皂苷在进样前需用0.22 μm有机滤膜过滤。

## 2 结果与讨论

### 2.1 固定相的表征

2.1.1 热重分析

采用热重分析仪对固定相进行热失重表征。从图2a可以看出,SiO_2_@HRHA和SiO_2_@DTP在200 ℃以下质量损失较少,说明这两种材料在200 ℃内具有较好的热稳定性;从200 ℃时开始分解到800 ℃时分解完全。而SiO_2_@AAE在200 ℃内质量损失较多,可能是由于含有的两个酯基在此温度范围内发生分解。SiO_2_@HRHA、SiO_2_@AAE和SiO_2_@DTP的键合量分别为8.55%、9.15%和6.40%。这3种固定相的热失重变化与SiO_2_-MAPS相比有明显差别,说明HRHA、AAE及DTP均已成功键合到SiO_2_-MAPS表面。

**图2:**
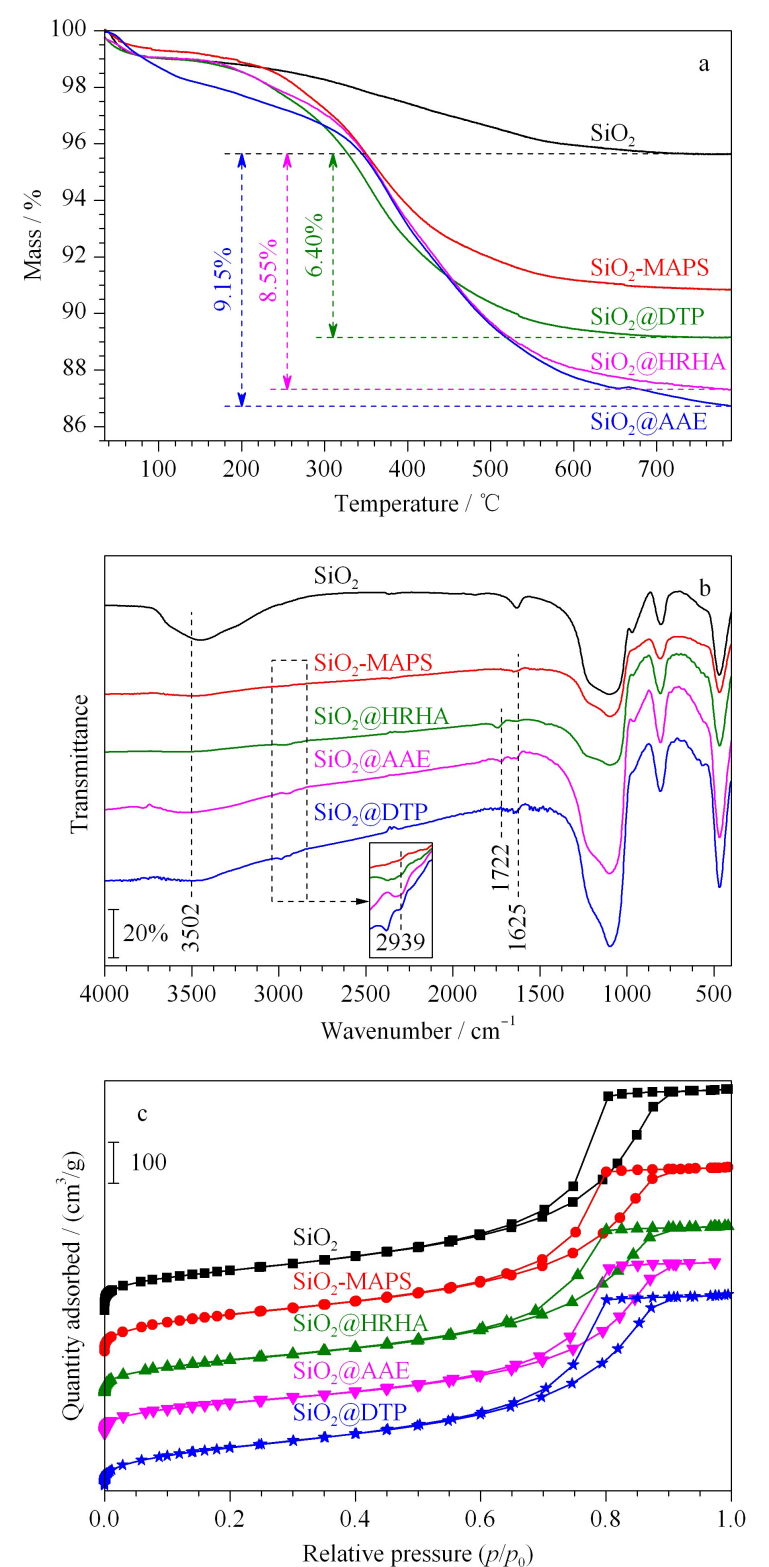
SiO_2_、SiO_2_-MAPS、SiO_2_@HRHA、SiO_2_@AAE和SiO_2_@DTP的(a)热重分析、(b)红外光谱及(c)氮气吸附-脱附等温线图

2.1.2 红外光谱

分别对SiO_2_、SiO_2_-MAPS、SiO_2_@HRHA、SiO_2_@AAE和SiO_2_@DTP进行傅立叶红外光谱测试,结果如图2b所示。3502 cm^-1^处为-OH特征吸收峰,1625 cm^-1^处是活化硅胶上结合水的伸缩振动峰,在2939 cm^-1^处为-CH_2_-和-CH_3_中C-H的伸缩振动峰。在SiO_2_-MAPS谱图中,3502 cm^-1^处峰值强度降低和2939 cm^-1^处特征峰的出现说明硅烷偶联剂已键合在硅胶表面;由于HRHA、AAE和DTP的引入使得SiO_2_@HRHA、SiO_2_@AAE和SiO_2_@DTP在2939 cm^-1^处的峰值强度增强,SiO_2_@HRHA和SiO_2_@AAE在1722 cm^-1^处有C=O伸缩振动峰的出现;SiO_2_@AAE和SiO_2_@DTP在3502 cm^-1^处的峰值由于AAE和DTP中-OH的存在而增大。结合热重表征的结果,可以确定SiO_2_@HRHA、SiO_2_@AAE和SiO_2_@DTP已制备成功。

2.1.3 孔结构表征

如图2c所示,与SiO_2_相比,固定相的氮气吸附-脱附曲线仍属于Ⅳ型等温线,属于介孔材料,没有改变硅胶的孔结构。由表1可知,与SiO_2_-MAPS相比,本研究制备的SiO_2_@HRHA、SiO_2_@AAE和SiO_2_@DTP比表面积、孔容和孔径均有所下降,但SiO_2_@HRHA仍有较大的比表面积和孔径可以满足其作为色谱材料的要求。

**表1:** SiO_2_、SiO_2_-MAPS、SiO_2_@HRHA、SiO_2_@AAE和SiO_2_@DTP的比表面积、孔容和孔径

Sample	Specific surface area/(m^2^/g)	Pore volume/ (cm^3^/g)	Average pore size/nm
SiO_2_	370.88	0.85	6.93
SiO_2_-MAPS	349.78	0.71	6.87
SiO_2_@HRHA	308.55	0.65	6.78
SiO_2_@AAE	290.05	0.64	6.24
SiO_2_@DTP	319.65	0.67	6.27

2.1.4 元素分析

SiO_2_、SiO_2_-MAPS和3种固定相的元素分析结果如表2所示。与SiO_2_相比,SiO_2_-MAPS中硫元素的出现,说明MAPS已成功键合在硅胶表面;与SiO_2_-MAPS相比,SiO_2_@HRHA、SiO_2_@AAE和SiO_2_@DTP固定相上碳和氢含量的增加证明了单体已成功键合到烷基化硅胶表面。SiO_2_@HRHA、SiO_2_@AAE和SiO_2_@DTP的表面覆盖度^[13]^分别为1.13、0. 86和1.16 μmol/m^2^。

**表2:** SiO_2_、SiO_2_-MAPS、SiO_2_@HRHA、SiO_2_@AAE和SiO_2_@DTP的元素分析结果

Sample	C/%	H/%	S/%
SiO_2_	1.77	0.48	0
SiO_2_-MAPS	3.61	1.15	2.13
SiO_2_@HRHA	6.08	2.30	1.22
SiO_2_@AAE	5.12	2.13	0.94
SiO_2_@DTP	5.43	1.93	1.26

### 2.2 色谱柱性能评价

2.2.1 流通性评价

以乙腈为流动相,通过改变不同的流速来测试SiO_2_@HRHA、SiO_2_@AAE和SiO_2_@DTP柱的流通性能。结果如图3所示,随着流速的增加,柱压逐渐升高;流动相的流速与柱压在3种色谱柱上表现出良好的线性关系,且相关系数(*R*^2^)均大于0.999。表明3种色谱柱流通性能良好,具有较好的耐压性能。

**图3:**
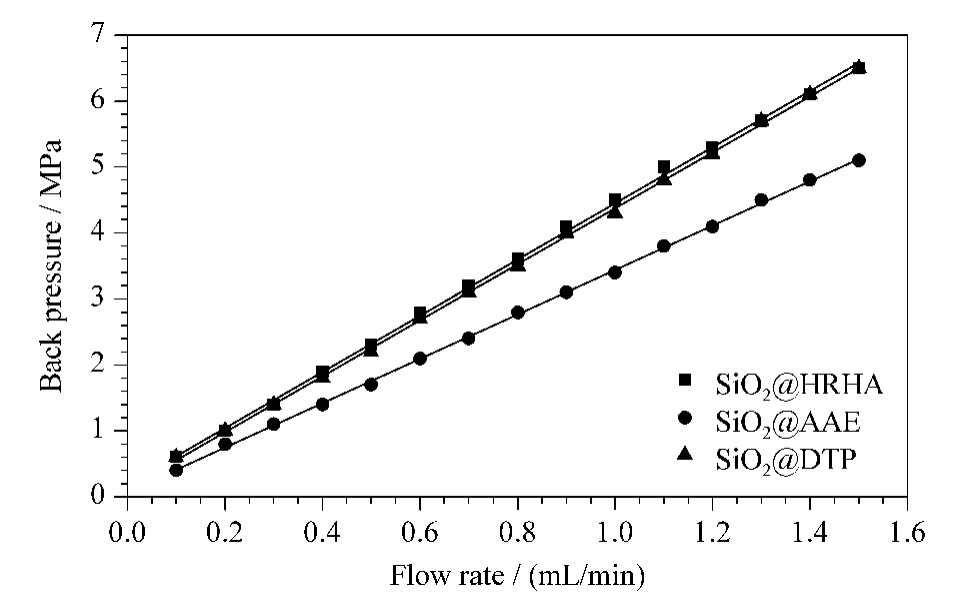
SiO_2_@HRHA、SiO_2_@AAE和SiO_2_@DTP柱的流速与柱压关系曲线

2.2.2 色谱柱的保留机制

以烷基苯为探针,在流速0.5 mL/min、波长254 nm的条件下考察3种色谱柱的保留机制。如图4所示,随着流动相中乙腈体积分数的增加,烷基苯在色谱柱上的保留时间不断下降,符合反相色谱的保留特征,说明这3种色谱柱均具备反相色谱性能。从图4d可知,烷基链的长度与烷基苯在色谱柱上的保留因子log *k*有很好的相关性,*R*^2^均为0.999;而线性回归曲线的斜率可以反映固定相与溶质之间疏水作用的强度^[14]^, SiO_2_@HRHA柱的线性拟合曲线的斜率值最大,表明SiO_2_@HRHA柱的疏水性优于SiO_2_@AAE柱和SiO_2_@DTP柱。

**图4:**
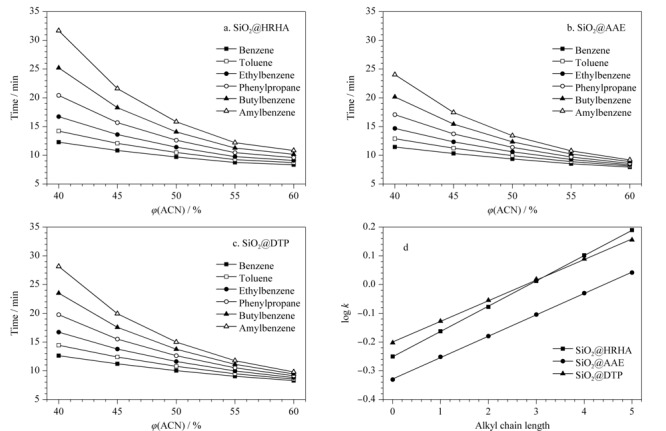
色谱柱反相色谱性能及疏水性能评价

2.2.3 重复性评价

重复性是评价色谱柱性能的一个重要指标。以苯、萘、芴、蒽、苯并菲为探针,在流速为0.5 mL/min、波长254 nm的条件下考察3种色谱柱的重复性。

将多环芳烃重复进样30次,依次选择第1、5、10、15、20、30次数据计算。通过计算可知,在SiO_2_@HRHA、SiO_2_@AAE和SiO_2_@DTP柱上溶质保留时间的相对标准偏差(RSD)分别为0.32%~0.66%、0.20%~0.49%及0.46%~1.21%,表明3种色谱柱均有较好的重复性。

2.2.4 Tanaka测试

色谱柱的Tanaka测试^[15]^是通过测试3种不同的混合物,根据其保留时间得到色谱柱的不同性能参数。本实验采用的混合物1由尿嘧啶、丁基苯、戊基苯、*o*-三联苯和苯并菲组成;混合物2由尿嘧啶、咖啡因和苯酚组成;混合物3由尿嘧啶、苯甲胺和苯酚组成,得出了疏水性、疏水选择性、立体选择性、氢键容量、pH>7和pH<3的离子交换容量。

如图5所示,SiO_2_@HRHA柱和SiO_2_@AAE柱的立体选择性*α*_T/O_值大于SiO_2_@DTP柱,说明SiO_2_@HRHA柱和SiO_2_@AAE柱具有较好的立体选择性,有利于平面和非平面化合物的分离^[16]^,这可能与HRHA和AAE中的三元类菲环骨架有关。

**图5:**
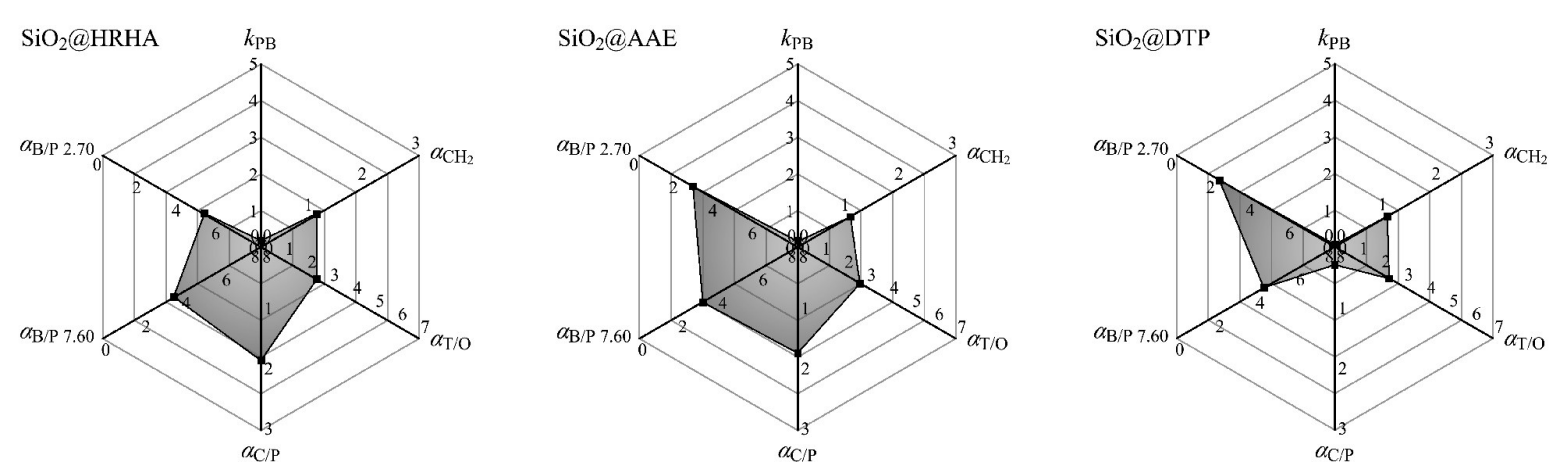
SiO_2_@HRHA、SiO_2_@AAE及SiO_2_@DTP柱的Tanaka测试参数图

SiO_2_@HRHA柱和SiO_2_@AAE柱的氢键容量*α*_C/P_值大于SiO_2_@DTP柱,可能是因为SiO_2_@HRHA柱和SiO_2_@AAE柱在硅胶表面的键合量大于SiO_2_@DTP柱,含有较多的酯基和羟基,能够与咖啡因形成氢键,增加溶质的保留,对极性溶质具有较好的分离选择性^[16,17]^。

### 2.3 三七总皂苷的分离

图6为4种色谱柱分别在各自最佳的色谱条件下分离三七总皂苷的色谱图,从图中可知,SiO_2_@HRHA柱和SiO_2_@AAE柱均有较好的分离效果,而SiO_2_@DTP柱分离效果不佳且出峰时间最长。其中SiO_2_@HRHA柱对三七总皂苷的综合分离效果最佳,分离度分别为3.33、3.54、20.17和9.72;并且对Rg1与Re的分离效果优于C18柱。SiO_2_@HRHA柱对三七总皂苷的分离效果优于SiO_2_@AAE和SiO_2_@DTP柱,这可能是由于SiO_2_@HRHA柱上松香基含有的三元类菲环骨架与多环化合物(三七总皂苷)具有结构相似性,有利于三七总皂苷的分离。除此之外,SiO_2_@HRHA柱较好的立体选择性和疏水性在三七总皂苷的分离过程中也发挥了一定作用。

**图6:**
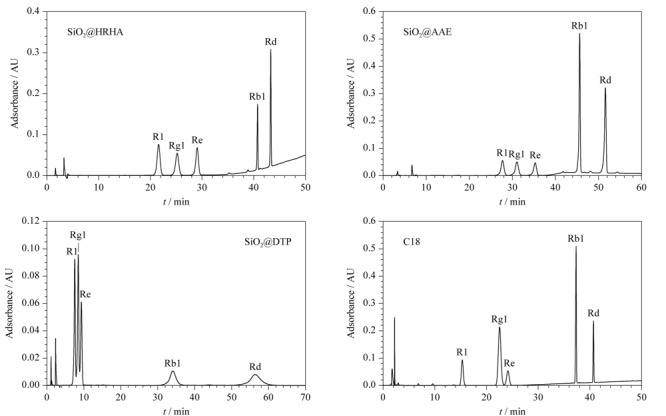
SiO_2_@HRHA、SiO_2_@AAE、SiO_2_@DTP和C18柱分离三七总皂苷

## 3 结论

本文将氢化松香丙烯酸羟乙酯键合到烷基化硅胶表面,成功制备了SiO_2_@HRHA固定相。填充的SiO_2_@HRHA柱具有典型的反相色谱保留机制、较好的立体选择性及氢键容量。结果表明,SiO_2_@HRHA柱对三七总皂苷具有较好的分离效果,说明改性松香键合二氧化硅高效液相色谱柱具有潜在的应用前景,为从实际样品中分离纯化三七总皂苷提供了新思路,为后续进一步研究该色谱柱对天然产物的分离性能奠定了基础。
